# Association between ApoA1 Gene, Plasma Lipid Profile, hsCRP Level, and Risk of Arterial Stiffness in Thai Elderly

**DOI:** 10.1155/2022/4930033

**Published:** 2022-07-14

**Authors:** Pruttaya Supajaree, Suwannee Chanprasertyothin, Prapimporn Chattranukulchai Shantavasinkul, Piyamitr Sritara, Jintana Sirivarasai

**Affiliations:** ^1^Master of Science Program in Nutrition, Faculty of Medicine Ramathibodi Hospital and Institute of Nutrition, Mahidol University, Bangkok, Thailand; ^2^Research & Innovation, Faculty of Medicine Ramathibodi Hospital, Mahidol University, Bangkok, Thailand; ^3^Department of Medicine, Faculty of Medicine Ramathibodi Hospital, Mahidol University, Bangkok, Thailand; ^4^Graduate Program in Nutrition, Faculty of Medicine Ramathibodi Hospital, Mahidol University, Bangkok, Thailand

## Abstract

**Introduction:**

Apolipoprotein A1 (ApoA1) gene polymorphism is linked to high-density lipoprotein cholesterol (HDL-C) levels. Variations in this gene, along with dyslipidemia and inflammation, may increase the risk of vascular stiffness. This study aimed to investigate the link between ApoA1 rs670 genetic variations, various biochemical parameters, and the risk of arterial stiffness in older people.

**Methods:**

This population-based cross-sectional study included 355 participants (≥60 years) who completed a demographic and lifestyle information questionnaire. Clinical and anthropometric examination, biochemical analysis, and ApoA1 rs670 genotyping by real-time PCR were performed. The cardio-ankle vascular index (CAVI) was used to assess arterial stiffness.

**Results:**

Age, BMI, waist circumference, SBP, LDL-C, and high-sensitivity C-reactive protein (hs-CRP) were associated with high CAVI (≥9) among older people. The mean CAVI (8.19 ± 2.78) for the ApoA1 rs670 AA genotype was lower than that of the GG genotypes (8.94 ± 1.00, *p* < 0.05). These results are supported by HDL-C (OR = 0.47, 95% CI: 0.24–0.93; *p*=0.030) and high hs-CRP (OR = 0.30, 95% CI: 0.16–0.57; *p*=0.006) levels together with adjusted ORs of both variables.

**Conclusion:**

ApoA1 rs670 genetic variations involved in the synthesis, transport, and processing of HDLs, hypertension, and inflammation are linked to arterial stiffness. Further studies are required to clarify these mechanisms.

## 1. Introduction

The prevalence of cardiovascular disease (CVD) increases with age in both men and women, as seen by the increasing percentage of CVD in people 40 to 60 years, 60 to 80 years, and over 80 years of age (35% to 40%, 75% to 78%, and over 85%, respectively) [1]. Arterial stiffness is an important predictor of CVD events and mortality with conventional CVD risk factors, including age, obesity, hypertension, hyperglycemia, and dyslipidemia. The cardio-ankle vascular index (CAVI) is a sensitive physiological index to evaluate arterial wall rigidity related to loss of arterial compliance [2]. Additionally, CAVI has been widely used clinically to assess arterial stiffness both in those with a known CVD risk and in those who are at risks such as older people and vulnerable groups with metabolic syndrome (MS), low-grade inflammatory state, smoking, drinking, and low physical activity [3].

Dyslipidemia is a conventional risk factor for arterial stiffness and is characterized by elevated total cholesterol (TC) or low-density lipoprotein cholesterol (LDL-C) levels and low levels of high-density lipoprotein cholesterol (HDL-C) or hypertriglyceridemia [4]. Mechanisms underlying dyslipidemia-induced arterial stiffness may involve increased inflammation. A previous study in 649 participants with dyslipidemia (median age, 66 years; range, 47.0–83.5 years) found a significant association between CAVI and age, systolic blood pressure (SBP), TC, LDL-C, and triglyceride (TG) levels [5]. Another study reported a relationship between arterial stiffness (evaluated by CAVI) and diabetes with a high TG-to-HDL cholesterol ratio and an odds ratio (OR) of 3.56 (95% confidence interval (CI): 1.50–8.46) for high TG-HDL diabetes [6]. Involvement of inflammation and arterial stiffness could be explained by high C-reactive protein (CRP) levels, which is a biomarker related to subclinical inflammatory states, and vascular inflammation. Its underlying mechanism involving CRP can induce abnormal physiological changes in the arterial wall by leucocytes, which release matrix metalloproteinases (MMPs) and break down elastin fibers [7].

In addition, to our knowledge, the first study conducted on the Thai population to evaluate the relationship between CAVI and coronary artery disease risk prediction showed that there was a correlation between CAVI and the prevalence of coronary stenosis after adjusting for traditional CAD risk factors (OR: 3.29). However, this study did not investigate the effect of genetic factors [8]. An integrated view of arterial stiffness involves both metabolic changes and genetic determinants. Genetic factors may indirectly or directly influence on the vascular wall through classical risk factors, leading to arterial stiffness. A candidate gene approach is proposed to identify important determinants of arterial stiffness, especially genes encoding proteins in the regulation of lipid metabolism. Apolipoprotein A1 (ApoA1) is the most abundant component of HDL-C, and genetic polymorphisms in the ApoA1 gene and their relationship with serum HDL cholesterol levels have been observed [9]. Another study provided evidence to support the role of the ApoA1 rs650 (75G > A) polymorphism and phenotype; the A allele was linked to significantly higher ApoA1 values in certain populations [10]. Several studies have investigated the impact of ApoA1 genetic polymorphisms on dyslipidemia and CVD risks, but the considerable effect of ApoA1 polymorphisms among different ethnic populations and their association with the risk of arterial stiffness in older people was limited. Therefore, this study was conducted to assess the effect of the AOPA1 rs 5650 polymorphism, dyslipidemia, and high sensitivity (hs)-CRP levels on arterial stiffness among older people.

## 2. Materials and Methods

### 2.1. Participants

The study population was 355 Thai older participants (60–80 years old) from the Electricity Generating Authority of Thailand (EGAT) cohort study. This project began in 1985, and participants are resurveyed every 5 years. Additionally, a study on gene-nutrient interactions was conducted in 2017. Details about the study design were previously described [11]. Inclusion criteria was participants aged 60 years and older, able to perform activities of daily living without assistance, and not hospitalized, and additionally, complete questionnaire data and serum or DNA for laboratory analysis. The exclusion criteria were as follows: those with cancer, severe hepatic or renal dysfunction, medical history of cardiovascular disease (i.e., atrial fibrillation, aortic stenosis, or aortic valve insufficiency), cardiopulmonary disease, or chronic inflammatory diseases. The self-administered questionnaires were used to collect data including general demographic data, lifestyle factors related to metabolic risks, history, family history, and use of medications. The project was approved by the ethics committee of the Faculty of Medicine Ramathibodi Hospital, Mahidol University (MU-RA2019/75), and all subjects signed the consent form. The protocol followed the recommendations for research on human beings as per the Declaration of Helsinki. Statement of Ethics.

### 2.2. Clinical and Biochemical Assessment

Physical examination, anthropometric measurements, and blood tests were also performed on the same day. Trained staff performed anthropometric measurements using standardized methods, body weight and height were determined, and the body mass index (BMI) was calculated as the weight (kg) divided by the height squared (m^2^). The waist circumference (WC) was measured to estimate central obesity or visceral adiposity, and it was assessed at the midpoint between the lower border of the rib cage and the iliac crest using a flexible measuring tape.

The CAVI, which is derived using the stiffness parameter obtained from this method, is theoretically unaffected by changes in blood pressure using the standardized method [12]. The mean CAVI value was automatically measured and calculated with VaSera VS-1000 (Fukuda Denshi, Tokyo, Japan) using methods described in previous studies [8, 12]. An increased CAVI represents pathological arterial stiffness, with a cut-off value of ≥9 [13]. The CAVI formula is shown in the following equation:

(1) $$\begin{array}{c} \text{CAVI}=a\left\{\left(\frac{2\rho }{\Delta p}\right)\times \;\ln\left(\frac{\text{Ps}}{\text{Pd}}\right){\text{PWV}}^{2}\right\}+b, \end{array}$$

where *a* and *b* are constants; Ps and Pd are SBP and DBP, respectively; *ρ* is blood density; Δ*p* is Ps-Pd; and PWV is the pulse wave velocity from the aortic valve to the ankle that eliminates the cardio-brachial path.

For biochemical measurements, participants fasted for 12 h overnight before venous blood samples were collected to determine the fasting plasma glucose (FPG), glycosylated hemoglobin A1C (HbA1C), TC, TG, HDL-C, LDL-C, hs-CRP, serum uric acid (SUA), alanine aminotransferase (ALT), aspartate aminotransferase (AST), and gamma-glutamyl transferase (GGT) levels. These tests were analyzed using automated methods (Cobas-Mira, Roche, Milan, Italy). The serum hs-CRP was measured using immunoassays (Immulite 1000 System; Siemens Health Diagnostics, Deerfield, IL, USA).

### 2.3. Genotyping for the ApoA1 Polymorphism

For the ApoA1 rs670 genotyping, DNA was extracted from peripheral blood leukocytes by proteinase K digestion and the phenol-chloroform method. ApoA1 rs670 (75G > A) polymorphism was analyzed by Taqman SNP allelic discrimination with the context sequence (VIC/FAM), which was GCTGGGAGGCTGATA AGCCCAGCCC[C/T] GGCCCTGTTGCTGCTCACTGGTCCT, using an ABI 7900H T (Applied Biosystems, Foster City, CA, USA) with the previous PCR-method described [14]. Further DNA sequencing was performed in 5% of samples (randomly selected from each genotype) for quality control.

### 2.4. Statistical Analyses

Statistical analysis was performed using SPSS for Windows version 23.0 (IBM, Inc., Armonk, NY, USA). Data are presented as the mean ± standard deviation (SD) for continuous variables and as the frequency (%) for categorical variables. Cigarette smoking was defined as smoking at least 1 cigarette/day for ≥1 year. Alcohol consumption was defined as drinking at least 500 g of alcohol/week for ≥1 year. The Kolmogorov–Smirnov test was used to determine the normality of continuous variables in this study, and the log transformation was conducted with skewed data. For hsCRP, data were not normally distributed and were transformed logarithmically, and the geometric mean was used. Continuous data were compared between two and three groups using Student's *t*-test and one-way analysis of variance, respectively. The Hardy–Weinberg equilibrium (HWE) was assessed using a goodness-of-fit chi-square test (*χ*^2^). Binary logistic regression analyses were used to evaluate the associated factors in the univariate and multivariate analyses. The parameters for this analysis were categorized on the basis of MS and MS components in accordance with the National Cholesterol Education Program Adult Treatment Panel III [15]. SBP ≥140 mmHg and DBP ≥90 mmHg were considered high blood pressure. Hyperglycemia was defined as FPG 126 mg/dL. Hypertriglyceridemia was defined as TG ≥150 mg/dL. Low HDL-C was defined as HDL-C <40 mg/dL for men and <50 for women. Central obesity was defined as WC ≥90 cm and ≥80 cm for Asian men and women, respectively [16]. Furthermore, previous research indicated that a body shape index (ABSI) with a cut-off value of 0.080 was associated with obesity-related metabolic disorders and arterial stiffness [17, 18]. ABSI formulation is calculated from this equation: WC/(BMI (2/3) height (1/2) [17]. High hs-CRP was defined as hs-CRP ≥3 mg/L which has been proposed for the assessment of risk for cardiovascular disease (CVD) [19]. Adjustments were made for the following potential confounding factors: sex, age, BMI, smoking, and drinking. The relationships are presented as the unadjusted OR and 95% CIs in the univariate analysis and as the adjusted OR (95% CI) in the multivariate analysis. Two-tailed *p* values <0.05 were considered statistically significant.

## 3. Results

This study included 355 participants (253 men, 72.28%; 102 women, 27.72%). No significant difference in age was observed between the male and female groups, and 56.9% and 54.4% of men were smokers and alcohol drinkers, respectively (Table 1). The mean BMI, WC, ABSI, TC, and HDL-C in women were significantly higher than those in men. Compared with female participants, male participants had higher blood pressure and elevated lipid profiles (TG and LDL-C), hs-CRP levels, and CAVI values.

CAVI is an indicator of arterial stiffness, with a cut-off value of ≥9 indicating a high risk of arterial stiffness [13]. We also evaluated the association between various metabolic risk factors among participants with CAVI <9 and ≥9 (Table 2). A high BMI (26.56 ± 3.48 vs. 24.22 ± 3.18 kg/m^2^), WC (92.76 ± 7.65 vs. 87.59 ± 8.92 cm), ABSI (0.081 ± 0.003 vs. 0.085 ± 0.004), and SBP (135.19 ± 19.70 vs. 130.00 ± 18.94 mmHg) were observed in participants with CAVI ≥9 compared with those with CAVI <9. Additionally, participants with CAVI ≥9 had a significantly higher hs-CRP level (2.65 ± 0.98 mg/L) than those with CAVI <9 (1.66 ± 0.78 mg/L, *p* < 0.05).

In our study, the ApoA1 rs650 genotype frequencies were 49.5% for GG, 44.5% for GA, and 6.0% for AA, with a minor allele frequency of 0.28. These genotype distributions were consistent with a population at HWE (chi-square value, 3.536; *p*=0.060). Individuals with an AA genotype had higher HDL-C but lower SBP, TG, hs-CRP, and CAVI values than those with a GG genotype (*p* < 0.05) (Table 3). No significant differences in other risk factors were observed among the three genotype groups.

Logistic regression analysis was performed after adjusting for sex, age, BMI, cigarette smoking, and alcohol drinking (Table 4). Significant predictors of arterial stiffness included high blood pressure (OR = 2.12, 95% CI: 1.43–3.16; *p*=0.001), hypertriglyceridemia (OR = 1.67, 95% CI: 1.06–2.61; *p*=0.024), low HDL-C (OR = 2.71, 95% CI: 1.46–4.98; *p*=0.001), and high hs-CRP (OR = 2.25, 95% CI: 1.33–3.80; *p*=0.022). After adjusting for these factors, the significant variables were high blood pressure, low HDL-C, and high hs-CRP. The effect of ApoA1 rs 670 (GG vs. GA + AA genotype) on risk of arterial stiffness showed only two significant variables associated with CAVI values, which were low HDL-C (OR = 0.47, 95% CI: 0.24–0.93; *p*=0.030) and high hs-CRP (OR = 0.30, 95% CI: 0.16–0.57; *p*=0.006, Table 5). The adjusted ORs for low HDL-C was 0.59 (95% CI: 0.24–0.88, *p*=0.041) and 0.28 for high hs-CRP (95% CI: 0.13–0.64; *p*=0.010).

## 4. Discussion

CAVI is a physiological surrogate marker of arterial stiffness, and an increased CAVI value may also directly promote other arterial pathologies. In this study, we found that the prevalence of arterial stiffness was 45.35% among older Thai people, which is similar to a previous study in older adults aged 60–89 years in which 47.8% of them had CAVI ≥9 [20]. More cases of arterial stiffness were found with increasing age, and the influence of gender has been reported with CVD risk factors. There was a cause and effect association between sex hormones and CVD, and female sex hormones were determined to be cardioprotective. There are several mechanisms that are involved in the development of arterial stiffness such as extracellular matrix alterations, vascular smooth muscle cell stiffening, endothelial dysfunction, renin-angiotensin-aldosterone system signaling, oxidative stress, and inflammation [21]. CAVI was both positively correlated with the endpoint of metabolic syndrome based on both WC and BMI [22]. Furthermore, ABSI was significantly associated with CAVI and the presence of MetS in the middle-aged population [23]. Previous studies have been also suggested ABSI as a more suitable clinical markers with arterial stiffening and potential useful for stratification of CVD risk [24, 25].

A previous longitudinal study showed that age and SBP were the main determinants of arterial stiffness [26]. Similar to our findings, individuals with CAVI ≥9 were significantly older and had a higher SBP than those with CAVI <9. The proposed mechanism of this vascular stiffening involves increasing age, which directly promoted MS, inflammation, and neurohormonal disorders. All these metabolic alterations resulted in endothelial dysfunction, increased MMPs and collagen, calcification, and decreased elastin, followed by the development of hypertension [27].

Inflammation also mediates arterial stiffness through changing the arterial wall structure. We found an association between CAVI and hs-CRP (Tables 2 and 4). A population-based cohort study including participants aged 55 years and older found an association between CRP and arterial stiffness [28]. Additionally, a significant relationship between increased CRP, MMP-9, and MMP-2 levels and systolic hypertension and arterial stiffness was demonstrated [7]. This mechanism is consistent with the evidence related to the upregulation of MMPs that are involved in aging-associated elastin fragmentation and collagen deposition [29]. With significant odds ratios and adjusted odds ratios for hypertriglyceridemia and low HDL-C, an increased risk of arterial stiffness was observed (Table 4). Previous studies found an association between different lipid parameters and aortic stiffness with significant ORs of non-HDL, TC/HDL, and TC [4]. The major cause of the lipid and lipoprotein abnormalities may be hypertriglyceridemia, which is a consequence of delayed TG-rich lipoprotein clearance and small dense LDL formation. Moreover, low HDL-C levels have been reported in obesity influenced by the dissociation of apo A-I from HDL, which results in lower HDL-C levels together with interference in reverse cholesterol transport and cellular cholesterol homeostasis [30].

ApoA1 rs 670 is a common G-to-A transition that is located at 75 base pairs (bp) upstream of the ApoA1 gene's transcription start point. Compared with 75G allele homozygotes, 75A allele carriers had higher transcription efficiency [31]. Variations in the ApoA1 gene could affect the protein function and quantity, HDL-C levels, and reverse cholesterol transport. ApoA1 rs670 effects on putative risk factors for arterial stiffness were investigated in this study. Several studies have confirmed these findings [32, 33]. Liao et al. reported that the ApoA1 75 A allele has a considerable effect on serum ApoA1 and HDL-C concentrations (*p* < 0.001) [32]. A study associating SNPs in the ApoA1 gene and obesity with the risk of low HDL-C disease showed that participants with the rs670 G allele had a 1.46 times (OR) greater risk of low HDL-C disease compared with other alleles (95% CI: 1.118–1.915; *p*=0.005) [33].

However, among the subgroup with arterial stiffness risk, only the GA + AA genotype was associated with a low risk related to HDL-C and hs-CRP levels (Table 5), which is consistent with a previous study. Both ApoA1 and HDL-C levels were significantly and inversely associated with hs-CRP (*p* < 0.01). HDL may enhance cholesterol export, reducing foam cell development and inhibiting endothelial cell adhesion molecule production in response to cytokines [34]. Furthermore, ApoA1 has anti-inflammatory properties that include downregulating the integrin expression to decrease immune cell transendothelial migration, inhibiting T-cell-induced monocyte activation and cytokine production, preventing lipid peroxidation, and interfering with innate immune receptors [35].

Our research has some limitations. Plasma ApoA1 concentrations were not measured, which makes it difficult to analyze the influence of rs670 on plasma ApoA1 levels in combination with HDL-C. Further study including plasma ApoA1 concentrations will provide more precise insights into the mechanisms underlying the elevated risk of arterial stiffness associated with this genetic variant. Because of the study's cross-sectional approach, no causal association can be established. Finally, although we adjusted for confounding factors such as gender, age, and lifestyle characteristics, other influencing factors may alter the study's results.

## 5. Conclusion

Our findings revealed a relationship between the A allele of the rs670 ApoA1 polymorphism, HDL-C, hs-CRP levels, and a lower risk of arterial stiffness in older people. Thus, ApoA1 genotyping could be useful in identifying individuals who are at high risk of arterial stiffness and suggesting strategies for preventing arterial stiffness linked to increased HDL-C levels. Further gene-gene interaction studies related to hypertension, the nitric oxide pathway, inflammation, and metalloproteinases may identify specific biological systems in the vascular wall.

## Figures and Tables

**Table 1:** General characteristic and biochemical parameters of the study population classified by gender (mean ± SD).

Parameters	Total (*N* = 355)	Gender		References
		Male (*N* = 253)	Female (*N* = 102)	
Age (years)	67.65 ± 4.11	67.73 ± 4.16	67.46 ± 4.02	—
Smoker, *n* (%)	149 (41.97)	144 (56.9)	5 (4.9)^a^	—
Drinker, *n* (%)	163 (41.97)	13 8 (54.5)	25 (24.5)^a^	—
BMI (kg/m^2^)	23.98 ± 3.14	23.92 ± 2.98	25.03 ± 4.59^a^	18.5–22.9
WC (cm)	87.16 ± 8.68	87.78 ± 8.51	85.63 ± 8.94^a^	≥90 (M); ≥80 (F)
ABSI	0.080 ± 0.003	0.080 ± 0.003	0.084 ± 0.004^a^	≤0.080
SBP (mmHg)	132.35 ± 19.43	133.77 ± 18.63	128.77 ± 20.99^a^	≤130
DBP (mmHg)	78.51 ± 11.23	79.50 ± 11.21	76.04 ± 11.11^a^	≤85
CAVI	8.47 ± 1.45	9.24 ± 1.65	8.92 ± 1.24^a^	<9
FBG (mg/dL)	91.40 ± 11.92	93.00 ± 12.75	87.42 ± 8.36^a^	<110
HbA1c (%)	5.77 ± 0.44	5.77 ± 0.48	5.74 ± 0.33^a^	≤6.5
TG (mg/dL)	115.11 ± 47.48	118.47 ± 48.89	106.95 ± 42.99^a^	<150
TC (mg/dL)	299.65 ± 40.95	224.99 ± 40.37	241.22 ± 40.26^a^	<200
HDL-C (mg/dL)	59.54 ± 16.27	56.69 ± 14.84	66.62 ± 17.51^a^	>40 (M), >50 (F)
LDL-C (mg/dL)	154.77 ± 33.73	153.65 ± 33.80	157.57 ± 33.54	<130
ALT (U/L)	20.19 ± 12.16	20.39 ± 10.42	19.96 ± 15.69	0–55
AST (U/L)	22.27 ± 7.67	22.17 ± 7.84	22.52 ± 7.25	5–34
GGT (U/L)	37.77 ± 42.13	39.26 ± 37.58	34.08 ± 51.77	5–61
BUN (mg/dL)	13.71 ± 3.21	13.84 ± 3.17	13.39 ± 3.32	9–20
Cr (mg/dL)	0.90 ± 0.16	0.98 ± 0.12	0.72 ± 0.10^a^	0.73–1.18
hs-CRP (mg/L)^*∗*^	1.76 ± 0.68	1.95 ± 0.69	1.55 ± 0.65	<3

^a^Significant difference from the male sex group, *p* < 0.05. ^*∗*^Geometric mean BMI, ABSI; a body shape index, body mass index; WC, waist circumference; SBP, systolic blood pressure; DBP, diastolic blood pressure; CAVI, cardio-ankle vascular index; FBG, fasting blood glucose; HbA1c, hemoglobin A1c; TG, triglycerides; TC, total cholesterol; HDL-C, high-density lipoprotein cholesterol; LDL-C, low-density lipoprotein cholesterol; ALT, alanine aminotransferase; AST, aspartate transferase; GGT, gamma-glutamyl transferase; BUN, blood urea nitrogen; Cr, creatinine; hs-CRP, high-sensitivity C-reactive protein; and SD, standard deviation.

**Table 2:** Various potential factors associated with CAVI (mean ± SD).

Parameters	CAVI	
	CAVI <9 (*N* = 194)	CAVI ≥9 (*N* = 161)
CAVI	8.20 ± 0.62	9.67 ± 0.95^a^
Age (years)	66.86 ± 3.76	68.61 ± 4.33^a^
BMI (kg/m^2^)	24.22 ± 3.18	26.56 ± 3.48^a^
WC (cm)	87.59 ± 8.92	92.76 ± 7.65^a^
ABSI	0.081 ± 0.003	0.085 ± 0.004^a^
SBP (mmHg)	130.00 ± 18.94	135.19 ± 19.70^a^
DBP (mmHg)	78.11 ± 10.96	79.00 ± 11.67
TC (mg/dL)	231.10 ± 41.01	225.50 ± 40.61
HDL-C (mg/dL)	61.04 ± 16.72	67.74 ± 9.56
LDL-C (mg/dL)	140.35 ± 32.90	152.63 ± 34.68^a^
TG (mg/dL)	116.02 ± 48.84	114.03 ± 45.94
FBG (mg/dL)	91.01 ± 10.44	91.87 ± 13.51
HbA1c (%)	5.75 ± 0.46	5.19 ± 0.42
hs-CRP (mg/L)^*∗*^	1.56 ± 2.05	2.35 ± 7.19^a^

^a^Significantly different from subjects with CAVI <9, *p* < 0.05. ^*∗*^Geometric mean. CAVI, cardio-ankle vascular index; BMI, body mass index; WC, waist circumference; ABSI; a body shape index, SBP, systolic blood pressure; DBP, diastolic blood pressure; TC, total cholesterol; HDL-C, high-density lipoprotein cholesterol; LDL-C, low-density lipoprotein cholesterol; TG, triglycerides; FBG, fasting blood glucose; HbA1c, hemoglobin A1c; hs-CRP, high-sensitivity C-reactive protein; SD, standard deviation.

**Table 3:** Biochemical variables (mean ± SD) of the study population classified by ApoA1 rs670 genotype.

Parameters	ApoA1 rs670		
	GG (*N* = 176)	GA (*N* = 158)	AA (*N* = 21)
Age (years)	67.92 ± 4.45	67.35 ± 3.68	67.67 ± 4.29
BMI (kg/m^2^)	24.17 ± 3.27	23.68 ± 2.96	24.54 ± 3.25
WC (cm)	87.86 ± 8.90	86.19 ± 9.27	88.56 ± 8.62
ABSI	0.080 ± 0.003	0.080 ± 0.003	0.081 ± 0.003
SBP (mmHg)	136.25 ± 12.33	131.74 ± 19.81	130.09 ± 15.54^a^
DBP (mmHg)	84.56 ± 10.86	77.80 ± 12.18	78.99 ± 6.52
CAVI	9.12 ± 3.56	8.94 ± 1.00	8.19 ± 2.78^a^
TC (mg/dL)	227.99 ± 40.89	230.62 ± 40.77	236.24 ± 43.87
HDL-C (mg/dL)	52.78 ± 11.98	61.06 ± 14.68	65.10 ± 15.55^a^
LDL-C (mg/dL)	154.41 ± 32.63	153.94 ± 34.36	150.69 ± 36.78
TG (mg/dL)	148.23 ± 36.99	128.56 ± 14.98	132.23 ± 16.33^a^
FBG (mg/dL)	91.96 ± 13.55	90.41 ± 9.83	94.24 ± 11.64
HbA1c (%)	5.78 ± 0.51	5.74 ± 0.38	5.80 ± 0.35
hs-CRP (mg/L)^*∗*^	2.51 ± 0.99	1.96 ± 0.74	1.94 ± 0.84^a^

^a^Significantly different from GG genotype, *p* < 0.05. ^*∗*^Geometric mean. ApoA1, apolipoprotein A1; BMI, body mass index; WC, waist circumference; ABSI; a body shape index, SBP, systolic blood pressure; DBP, diastolic blood pressure; CAVI, cardio-ankle vascular index; TC, total cholesterol; HDL-C, high-density lipoprotein cholesterol; LDL-C, low-density lipoprotein cholesterol; TG, triglycerides; FBG, fasting blood glucose; HbA1c, hemoglobin A1c; hs-CRP, high-sensitivity C-reactive protein; SD, standard deviation.

**Table 4:** Associations between the CAVI value and metabolic risk factors.

		CAVI	
		Crude OR (95% CI)	Adjusted OR (95% CI)^a^
Metabolic syndrome	No	Reference	Reference
	Yes	1.77 (1.10–2.85)	1.52 (0.92–2.51)
	*p* value	0.19	0.102

Central obesity	No	Reference	Reference
	Yes	1.27 (0.85–1.86)	1.36 (0.89–2.07)
	*p* value	0.230	0.151

High blood pressure	No	Reference	Reference
	Yes	2.12 (1.43–3.16)	2.07 (1.36–3.17)
	*p* value	0.001	0.001

Hyperglycemia	No	Reference	Reference
	Yes	1.39 (0.79–2.23)	1.41 (0.83–2.41)
	*p* value	0.280	0.195

Hypertriglyceridemia	No	Reference	Reference
	Yes	1.67 (1.06–2.61)	1.57 (0.98–2.50)
	*p* value	0.024	0.55

Low HDL-C	No	Reference	Reference
	Yes	2.71 (1.46–4.98)	2.96 (1.54–5.69)
	*p* value	0.001	0.001

High hs-CRP level^b^	No	Reference	Reference
	Yes	2.25 (1.33–3.80)	2.57 (1.45–4.52)
	*p* value	0.022	0.001

^a^Adjusted OR calculated from the logistic regression adjusted for sex, age, BMI, smoking, and drinking. ^b^Geometric mean of the hs-CRP level. CAVI, cardio-ankle vascular index; OR, odds ratio; CI, confidence interval; HDL-C, high-density lipoprotein cholesterol; hs-CRP, high-sensitivity C-reactive protein.

**Table 5:** Association between the ApoA1 polymorphism related to metabolic components and risk of arterial stiffness on the basis of the CAVI index.

		Crude OR (95% CI)	Adjusted OR (95% CI)^a^
Metabolic syndrome	GG	Reference	Reference
	GA + AA	0.54 (0.28–1.04)	0.79 (0.36–1.46)
	*p* value	0.681	0.375

Central obesity	GG	Reference	Reference
	GA + AA	0.72 (0.39–1.32)	0.91 (0.47–1.76)
	*p* value	0.298	0.792

High blood pressure	GG	Reference	Reference
	GA + AA	1.52 (0.83–2.78)	1.85 (0.94–3.67)
	*p* value	0.173	0.740

Hyperglycemia	GG	Reference	Reference
	GA + AA	0.69 (0.34–1.42)	0.90 (0.42–1.91)
	*p* value	0.323	0.791

Hypertriglyceridemia	GG	Reference	Reference
	GA + AA	1.09 (0.52–1.93)	1.12 (0.55–2.27)
	*p* value	0.978	0.737

Low HDL-C	GG	Reference	Reference
	GA + AA	0.474 (0.24–0.93)	0.59 (0.24–0.88)
	*p* value	0.030	0.041

High hs-CRP level^b^	GG	Reference	Reference
	GA + AA	0.30 (0.16–0.57)	0.28 (0.13–0.64)
	*p* value	0.006	0.010

^a^Adjusted OR calculated from the logistic regression adjusted for sex, age, BMI, smoking, and drinking. ^b^Geometric mean of the hs-CRP level. ApoA1, apolipoprotein A1; CAVI, cardio-ankle vascular index; OR, odds ratio; CI, confidence interval; HDL-C, high-density lipoprotein cholesterol; hs-CRP, high-sensitivity C-reactive protein.

## Data Availability

All data are included within the manuscript, and further inquiries can be directed to the corresponding authors.

## References

[1] Benjamin E. J., Muntner P., Alonso A. (2019). Heart disease and stroke statistics-2019 update: a report from the American heart association. *Circulation*.

[2] Shirai K., Hiruta N., Song M. (2011). Cardio-ankle vascular index (CAVI) as a novel indicator of arterial stiffness: theory, evidence and perspectives. *Journal of Atherosclerosis and Thrombosis*.

[3] Saiki A., Sato Y., Watanabe R. (2016). The role of a novel arterial stiffness parameter, cardio-ankle vascular index (CAVI), as a surrogate marker for cardiovascular diseases. *Journal of Atherosclerosis and Thrombosis*.

[4] Vallée A., Lelong H., Lopez-Sublet M., Topouchian J., Safar M. E., Blacher J. (2019). Association between different lipid parameters and aortic stiffness: clinical and therapeutic implication perspectives. *Journal of Hypertension*.

[5] Zhao X., Bo L., Zhao H., Li L., Zhou Y., Wang H. (2018). Cardio-ankle vascular index value in dyslipidemia patients affected by cardiovascular risk factors. *Clinical and Experimental Hypertension*.

[6] Shimizu Y., Nakazato M., Sekita T. (2013). Association of arterial stiffness and diabetes with triglycerides-to-HDL cholesterol ratio for Japanese men: the Nagasaki islands study. *Atherosclerosis*.

[7] Yasmin, McEniery C. M., Wallace S. (2005). Matrix metalloproteinase-9 (MMP-9), MMP-2, and serum elastase activity are associated with systolic hypertension and arterial stiffness. *Arteriosclerosis, Thrombosis, and Vascular Biology*.

[8] Yingchoncharoen T., Limpijankit T., Jongjirasiri S., Laothamatas J., Yamwong S., Sritara P. (2012). Arterial stiffness contributes to coronary artery disease risk prediction beyond the traditional risk score (RAMA-EGAT score). *Heart Asia*.

[9] Bandarian F., Hedayati M., Daneshpour M. S., Naseri M., Azizi F. (2013). Genetic polymorphisms in the APOA1 gene and their relationship with serum HDL cholesterol levels. *Lipids*.

[10] Dawar R., Gurtoo A., Singh R. (2010). Apolipoprotein A1 gene polymorphism (G-75A and C+83T) in patients with myocardial infarction: a pilot study in a north Indian population. *American Journal of Clinical Pathology*.

[11] Vathesatogkit P., Woodward M., Tanomsup S. (2012). Cohort profile: the electricity generating authority of Thailand study. *International Journal of Epidemiology*.

[12] Shirai K., Utino J., Otsuka K., Takata M. (2006). A novel blood pressure-independent arterial wall stiffness parameter; cardio-ankle vascular index (CAVI). *Journal of Atherosclerosis and Thrombosis*.

[13] Sun C. K. (2013). Cardio-ankle vascular index (CAVI) as an indicator of arterial stiffness. *Integrated Blood Pressure Control*.

[14] Torres N., Guevara-Cruz M., Granados J. (2009). Reduction of serum lipids by soy protein and soluble fiber is not associated with the ABCG5/G8, apolipoprotein E, and apolipoprotein A1 polymorphisms in a group of hyperlipidemic Mexican subjects. *Nutrition Research*.

[15] Grundy S. M., Cleeman J. I., Daniels S. R. (2005). American heart association; national heart, lung, and blood institute. diagnosis and management of the metabolic syndrome: an American heart association/national heart, lung, and blood institute scientific statement. *Circulation*.

[16] World Health Organization (1997). *Obesity: Preventing and Managing the Global Epidemic*.

[17] Krakauer N. Y., Krakauer J. C. (2012). A new body shape index predicts mortality hazard independently of body mass index. *PLoS One*.

[18] Nagayama D., Watanabe Y., Yamaguchi T. (2020). New index of abdominal obesity, a body shape index, is BMI-independently associated with systemic arterial stiffness in real-world Japanese population. *International Journal of Clinical Pharmacology & Therapeutics*.

[19] Ridker P. M., Cook N. (2004). Clinical usefulness of very high and very low levels of C-reactive protein across the full range of framingham risk scores. *Circulation*.

[20] Turusheva A., Frolova E., Kotovskaya Y., Petrosyan Y., Dumbadze R. (2020). Association between arterial stiffness, frailty and fall-related injuries in older adults. *Vascular Health and Risk Management*.

[21] Du Pont J. J., Kenney R. M., Patel A. R., Jaffe I. Z. (2019). Sex differences in mechanisms of arterial stiffness. *British Journal of Pharmacology*.

[22] Sugiura T., Dohi Y., Takagi Y. (2020). Relationships of obesity-related indices and metabolic syndrome with subclinical atherosclerosis in middle-aged untreated Japanese workers. *Journal of Atherosclerosis and Thrombosis*.

[23] Kim S., Choi S. Y., Lee H., Kim J. J., Park H. E. (2022). Sex and age differences in the impact of metabolic syndrome and its components including a body shape index on arterial stiffness in the general population. *Journal of Atherosclerosis and Thrombosis*.

[24] Sugiura T., Dohi Y., Takagi Y. (2021). A body shape index could serve to identify individuals with metabolic syndrome and increased arterial stiffness in the middle-aged population. *Clinical Nutrition ESPEN*.

[25] Nagayama D., Watanabe Y., Yamaguchi T. (2022). Issue of waist circumference for the diagnosis of metabolic syndrome regarding arterial stiffness: possible utility of a body shape index in middle-aged nonobese Japanese urban residents receiving health screening. *Obesity Facts*.

[26] Al Ghatrif M., Strait J. B., Morrell C. H. (2013). Longitudinal trajectories of arterial stiffness and the role of blood pressure: the Baltimore longitudinal study of aging. *Hypertension*.

[27] Sun Z. (2015). Aging, arterial stiffness, and hypertension. *Hypertension*.

[28] Mattace-Raso F. U., Van Der Cammen T. J., Van Der Meer I. M. (2004). C-reactive protein and arterial stiffness in older adults: the rotterdam study. *Atherosclerosis*.

[29] Wang M., Zhang J., Telljohann R. (2012). Chronic matrix metalloproteinase inhibition retards age-associated arterial proinflammation and increase in blood pressure. *Hypertension*.

[30] Klop B., Elte J. W., Cabezas M. C. (2013). Dyslipidemia in obesity: mechanisms and potential targets. *Nutrients*.

[31] Wang X. L., Badenhop R. B., Sim A. S., Wilcken D. E. L. (1998). The effect on transcription efficiency of the apolipoprotein AI gene of DNA variants at the 5’ untranslated region. *International Journal of Clinical and Laboratory Research*.

[32] Liao B., Cheng K., Dong S., Liu H., Xu Z. (2015). Effect of apolipoprotein A1 genetic polymorphisms on lipid profiles and the risk of coronary artery disease. *Diagnostic Pathology*.

[33] Wang X., He J., Guo H. (2017). Interactions of six SNPs in APOA1 gene and types of obesity on low HDL-C disease in Xinjiang pastoral area of China. *Lipids in Health and Disease*.

[34] Xu W., Li R., Zhang S. (2012). The relationship between high-sensitivity C-reactive protein and ApoB, ApoB/ApoA1 ratio in general population of China. *Endocrine*.

[35] Vuilleumier N., Dayer J. M., Von Eckardstein A., Roux-Lombard P. (2013). Pro- or anti-inflammatory role of apolipoprotein A-1 in high-density lipoproteins?. *Swiss Medical Weekly*.

